# Unveiling the kinetic versatility of aryl-alcohol oxidases with different electron acceptors

**DOI:** 10.3389/fbioe.2024.1440598

**Published:** 2024-08-05

**Authors:** Ana Serrano, Paula Cinca-Fernando, Juan Carro, Adrián Velázquez-Campoy, Marta Martínez-Júlvez, Ángel T. Martínez, Patricia Ferreira

**Affiliations:** ^1^ Centro de Investigaciones Biológicas Margarita Salas, CSIC, Madrid, Spain; ^2^ Departamento de Bioquímica y Biología Molecular y Celular, Facultad de Ciencias, Universidad de Zaragoza, Zaragoza, Spain; ^3^ Instituto de Biocomputación y Física de Sistemas Complejos, BIFI (GBsC-CSIC Joint Unit), Universidad de Zaragoza, Zaragoza, Spain; ^4^ Institute for Health Research Aragon (IIS Aragon), Zaragoza, Spain; ^5^ Biomedical Research Networking Center in Hepatic and Digestive Diseases (CIBERehd), Madrid, Spain

**Keywords:** aryl-alcohol oxidases (AAO), glucose-methanol-choline oxidase/dehydrogenase (GMC) superfamily, molecular oxygen reduction, quinone reduction, lignocellulose decay, structural-functional properties, catalytic mechanism

## Abstract

**Introduction:** Aryl-alcohol oxidase (AAO) shows a pronounced duality as oxidase and dehydrogenase similar to that described for other glucose-methanol-choline (GMC) oxidase/dehydrogenase superfamily proteins involved in lignocellulose decomposition. In this work, we detail the overall mechanism of AAOs from *Pleurotus eryngii* and *Bjerkandera adusta* for catalyzing the oxidation of natural aryl-alcohol substrates using either oxygen or quinones as electron acceptors and describe the crystallographic structure of AAO from *B. adusta* in complex with a product analogue.

**Methods:** Kinetic studies with 4-methoxybenzyl and 3-chloro-4- methoxybenzyl alcohols, including both transient-state and steady-state analyses, along with interaction studies, provide insight into the oxidase and dehydrogenase mechanisms of these enzymes. Moreover, the resolution of the crystal structure of AAO from *B. adusta* allowed us to compare their overall folding and the structure of the active sites of both AAOs in relation to their activities.

**Results and Discussion:** Although both enzymes show similar mechanistic properties, notable differences are highlighted in this study. In *B. adusta*, the AAO oxidase activity is limited by the reoxidation of the flavin, while in *P. eryngii* the slower step takes place during the reductive half-reaction, which determines the overall reaction rate. By contrast, dehydrogenase activity in both enzymes, irrespective of the alcohol participating in the reaction, is limited by the hydroquinone release from the active site. Despite these differences, both AAOs are more efficient as dehydrogenases, supporting the physiological role of this activity in lignocellulosic decay. This dual activity would allow these enzymes to adapt to different environments based on the available electron acceptors.

## 1 Introduction

Fungal decay of lignocellulose plays an essential role in the carbon cycle since lignocellulosic materials, primarily composed of cellulose, hemicellulose and lignin, constitute an enormous carbon reservoir (Bugg, 2024). While cellulose and hemicelluloses are easily degraded by hydrolytic enzymes, lignin is a recalcitrant aromatic polymer whose degradation involves an extracellular oxidative multi-enzymatic system (Janusz et al., 2017; Andlar et al., 2018). Lignin can be degraded either by laccases, using molecular oxygen (O_2_), or by lignin peroxidases, manganese peroxidases and versatile peroxidases, which are activated by hydrogen peroxide (Fabbri et al., 2023). Not only is the latter crucial for this reaction, but also for the non-enzymatic attack of lignocelluloses by reactive oxygen species (Castaño et al., 2018). Hydrogen peroxide is generated by accessory enzymes such as glyoxal oxidases, copper-radical oxidases and flavoenzymes from the glucose-methanol-choline oxidase/dehydrogenase (GMC) superfamily (Sützl et al., 2018). GMC enzymes involved in lignocellulose degradation include pyranose 2-oxidases (P2O), cellobiose dehydrogenases, methanol oxidases and aryl-alcohol oxidases (AAO), among others (Cavener, 1992; Sützl et al., 2019).

AAOs are flavin adenine dinucleotide (FAD)-containing proteins capable of catalyzing the oxidative dehydrogenation of nonphenolic and phenolic aryl alcohols, heterocyclic alcohols, as well as polyunsaturated aliphatic alcohols, into their corresponding aldehydes (Chakraborty et al., 2014; Couturier et al., 2016; Tamaru et al., 2018; Lappe et al., 2021; Urlacher and Koschorreck, 2021). This enzyme has been widely characterized in *Pleurotus eryngii* (*Pe*AAO), a basidiomycete able to selectively remove lignin. The active site of AAOs is enclosed from the outer environment by an insertion that creates a loop over the active-site channel (Cavener, 1992). Furthermore, structural analysis and molecular dynamics performed on *Pe*AAO have revealed that its active site is only accessible through a narrow hydrophobic channel (Fernández et al., 2009; Hernández-Ortega et al., 2011), characteristic of the AAO family, unlike other GMCs where the access to the active site is wide and open (Andlar et al., 2018). Due to this narrow gate, Phe397 and Tyr92 side chains must reorganize in order to allow alcohol substrates to access the active site (Ferreira et al., 2015; Carro et al., 2018a).

The catalytic mechanism of *Pe*AAO is split into two sequential semi-reactions. In the reductive-half reaction, the alcohol is two-electron oxidized in a concerted reaction involving a hydride transfer to the flavin N5 atom and the abstraction of a proton by the catalytic base His502 assisted by His546, resulting in flavin reduction. In the oxidative-half reaction, reduced FAD reacts with O_2_, which freely diffuses to the active site, to yield superoxide anion radical and the neutral semiquinone, which is not thermodynamically stabilized. Flavin oxidation is completed through the transfer of a hydrogen atom from the flavin N5 atom to superoxide and an exchangeable proton from His502 or a solvent exchangeable site (Ferreira et al., 2009; Hernández-Ortega et al., 2012; Carro et al., 2018b). In addition to this well-characterized oxidase activity of AAOs, a simultaneous quinone-reductase activity has been reported recently in *Pe*AAO and its homologous enzymes from *P. ostreatus* and *Bjerkandera adusta* (Ferreira et al., 2023), as well as in *Ustilago maydis* AAO (Couturier et al., 2016). Such a dual oxidase/dehydrogenase activity has been previously described in other members of the GMC superfamily, as glucose oxidase (GOX) and P2O (Leskovac et al., 2005; Herzog et al., 2019; Mtemeri and Hickey, 2023). In the above AAOs, quinone reductase activity is similar or even higher, in terms of catalytic efficiencies, than the oxidase activity, indicating that both O_2_ and quinones may act as natural oxidizing substrates, leading to competition when both are present. Moreover, these classical AAO enzymes differ from the reported aryl-alcohol dehydrogenases (AAQOs) from *Pycnoporus cinnabarinus*, which exhibit a preferential quinone reductase activity compared to their insignificant oxidase activity (Mathieu et al., 2016). These findings reinforce the idea that the AAO quinone-reducing activity may play a physiological role in lignin biodegradation.

In this paper, we present a comparative study on the mechanistic properties of AAOs as oxidases and dehydrogenases, using the enzymes from *B. adusta* (*Ba*AAO) and *P. eryngii* as models. We investigate the oxidation of two compounds, 4-methoxybenzyl and 3-chloro-4-methoxybenzyl alcohols, which are physiologically secreted by fungi from these two species (Gutiérrez et al., 1994; Silk et al., 2001). For the first time, these oxidase and quinone-reductase activities are studied by combining steady-state and transient-state kinetics, turnover studies and isothermal titration calorimetry, aiming to elucidate the global catalytic mechanism and limiting steps involved in the alcohol oxidation by AAOs using either O_2_ or 1,4-benzoquinone as electron acceptors. Additionally, we have solved the crystallographic structure of *Ba*AAO in complex with a product analogue, which complements the existing structures of *Pe*AAO and provides insight into the access channels and active sites in AAO enzymes.

## 2 Material and methods

### 2.1 Chemicals

4-methoxybenzyl alcohol, 4-methoxybenzoic acid, 3-Cl-4-methoxybenzoic acid and 1,4-benzoquinone (BQ) were obtained from Sigma-Aldrich. 3-chloro-4-methoxybenzyl alcohol was synthesized at the Instituto de Nanociencia y Ciencia de Materiales de Aragón (CSIC-UZ, Zaragoza, Spain).

### 2.2 Heterologous production of AAOs in *E. coli* and *in vitro* activation

Recombinant *Pe*AAO and *Ba*AAO were obtained by *Escherichia coli* expression of the mature AAO cDNAs (GenBank AF064069 and JGI 171002, respectively) followed by *in vitro* refolding and activation in the presence of the cofactor, and purification by ion-exchange chromatography as previously described (Ruiz-Dueñas et al., 2006; Ferreira et al., 2023).

### 2.3 Steady-state kinetics

Steady-state kinetic parameters were measured spectrophotometrically by monitoring the oxidation of 4-methoxybenzyl and 3-chloro-4-methoxybenzyl alcohols to their respective aldehydes in 50 mM sodium phosphate at pH 6.0 and 25 °C.

Bi-substrate kinetics were analyzed by concurrently varying the concentration of the aforementioned alcohols and electron acceptors. Oxygen assays were carried out in a screw-cap cuvette where the alcohol was equilibrated with the desired concentration of O_2_ by bubbling with the appropriate O_2_/N_2_ gas mixture (4%, 10%, 21%, 44%, and 100% O_2_) for 10 min. Alcohol oxidation was initiated by the addition of the enzyme and followed at 285 nm for 4-methoxybenzaldehyde production and 295 nm in the case of 3-Cl-4-methoxybenzaldehyde (Δε_285_ = 16,950 M^−1^cm^−1^ and Δε_295_ = 15,000 M^−1^cm^−1^, respectively) (Ferreira et al., 2005). Benzoquinone (BQ) was selected as electron acceptor to test dehydrogenase activity, which was measured in screw cap cuvettes after O_2_ removal by Ar bubbling for 10 min. BQ reduction was monitored at 247 nm using the extinction coefficients of 17,833 M^−1^cm^−1^ and 18,067 M^−1^cm^−1^ in 4-methoxybenzyl alcohol and 3-Cl-4-methoxybenzyl alcohol reactions, respectively. These extinction coefficients were previously determined, taking into account the Δε_247_ = 20,200 M^−1^cm^−1^ for the reduction of BQ to hydroquinone (Wilcoxen et al., 2011) and the corresponding absorptivity of each aldehyde at this wavelength. BQ solutions were prepared immediately prior to use and protected from the light to avoid photodegradation.

The initial rates for bi-substrate kinetics were fitted to Eq. 1 or Eq. 2, which describe sequential and ping-pong kinetic mechanisms, respectively. In these equations, *e* represents the enzyme concentration, *k*_cat_ is the maximum turnover, *A* is the alcohol concentration, *B* is the electron acceptor concentration, $K_{m}^{A}$ and $K_{m}^{B}$ are the Michaelis constants for *A* and *B* respectively, and $K_{d}$ is the dissociation constant for the alcohol substrate:

$$\frac{v_{0}}{e}=\frac{k_{cat}\left[A\right]\left[B\right]}{K_{m}^{B}\left[A\right]+K_{m}^{A}\left[B\right]+\left[A\right]\left[B\right]+K_{d}K_{m}^{B}}$$ (1)

$$\frac{v_{0}}{e}=\frac{k_{cat}\left[A\right]\left[B\right]}{K_{m}^{B}\left[A\right]+K_{m}^{A}\left[B\right]+\left[A\right]\left[B\right]}$$ (2)

Additionally, Hanes–Woolf graphical representation ([A]/*v*_0_ vs. [A]) was used to discriminate between sequential (parallel lines) and ping-pong (intersecting lines) mechanisms.

### 2.4 Stopped-flow measurements: enzyme turnover and pre-steady-state kinetics

Stopped-flow experiments were performed using an SX18.MV stopped-flow spectrophotometer (Applied Photophysics Ltd., Surrey, United Kingdom) interfaced with the ProData-SX software and a monochromator or a photodiode array detector. Assays were carried out in 50 mM sodium phosphate, pH 6.0 at 25 °C, unless stated otherwise.

Whenever anaerobiosis was necessary, all buffers, substrates and the enzyme were placed into glass tonometers that were subjected to 20–25 cycles of evacuation and argon flushing to remove O_2_. To ensure anaerobiosis, glucose (10 mM) and GOX (10 UmL^−1^) were added after some vacuum-argon cycles. Besides, the stopped-flow equipment was made anaerobic by flushing a solution of sodium dithionite previously subjected to 10–15 cycles of vacuum-argon. Afterward, the sodium dithionite solution was removed by rinsing the equipment with anaerobic buffer. Stopped-flow measurements were then conducted after establishing a baseline with the buffer.

For enzyme-turnover experiments, saturating alcohol concentrations (at least 10-fold the *K*_m_) were mixed with AAO (∼10 μM, final concentration) in the presence of either O_2_ or BQ. Experiments with O_2_ were performed with the alcohol under air-saturated conditions, while in the case of BQ, the alcohol was mixed with saturating concentration of BQ (300 µM) under anaerobic conditions. The spectral evolution of the enzymes during redox turnover was recorded between 350 and 700 nm.

Studies on the reductive half-reaction were performed by mixing AAO (∼10 μM, final concentration) with increasing concentrations of alcohols under anaerobic conditions. Observed rate constants (*k*_obs_) were calculated using global analysis and numerical integration methods, simultaneously utilizing all spectral data in the 400–800 nm region along time evolution using the ProKineticist software from Applied Photophysics. A single-step model (A → B) best fitted the overall reaction at all alcohol concentrations assayed. Averaged *k*_obs_ values from 3 to 5 replicates at each substrate concentration were then fitted to Eq. 3, describing the formation of an enzyme:substrate complex prior to the flavin reduction, where *k*_red_ and *k*_rev_ represent the reduction rate constant at infinite substrate concentration and its reverse reaction, respectively; *A* represents the alcohol concentration; and *K*_d_ is the alcohol dissociation constant.

$$k_{obs}=\frac{k_{red}\left[A\right]}{K_{d\left(A\right)}+\left[A\right]}+k_{rev}$$ (3)

The oxidative half-reaction was investigated by mixing reduced AAO (∼10 μM, final concentration) with increasing O_2_ or BQ concentrations in 50 mM sodium phosphate, pH 6.0 at 12 °C. The enzyme was reduced with a 1.5-fold excess of either 4-methoxybenzyl or 3-Cl-4-methoxybenzyl alcohols in anaerobic conditions as previously described (Ferreira et al., 2015). *k*_obs_ for the flavin reoxidation were obtained by either global fitting of the spectra or fitting the monochromator traces to exponential equations describing one-step or two-step processes (A → B and A → B → C). Averaged *k*_obs_ of 3–5 replicates were fitted to Eq. 4 that describes a linear dependence on the electron acceptor concentration to estimate the apparent second-order rate constant for reoxidation (^app^ *k*_ox_):

$$k_{obs}{=}^{app}k_{ox}\left[acceptor\right]+k_{rev}$$ (4)

Estimation of the rates of the AAO:4-methoxybenzoic acid and AAO:3-Cl-4-methoxybenzoic acid complex formation and dissociation were performed by analyzing spectral changes upon mixing enzyme (∼10 µM) with different concentrations of the ligand in 50 mM sodium phosphate, pH 6.0 at 12 °C. Data were globally fitted to an equation describing a one-step process. The obtained *k*_obs_ were linear functions of the ligand concentration and were fitted to Eq. 5, where *k*_on_ stands for the second-order rate constant for the complex formation; [L] is the ligand concentration, and *k*_off_ is the rate constant for the complex dissociation.

$$k_{obs}=k_{on}\left[L\right]+k_{off}$$ (5)

### 2.5 Isothermal titration calorimetry (ITC)

The interaction parameters of the enzymes with BQ were determined using an Auto-ITC200 high-sensitivity microcalorimeter (MicroCal, Malvern-Panalytical) thermostatted at 25 °C. Enzyme solutions (∼6 μM for *Ba*AAO and 15 μM for *Pe*AAO) were titrated with BQ (∼50 µM for *Ba*AAO and ∼100 μM for *Pe*AAO) in 50 mM Tris/HCl, pH 7.0. Up to 19 injections of 2 µL were programmed with enough time spacing for the signal to recover the baseline. The association constant (*K*_a_), the enthalpy change (Δ*H*) and the apparent stoichiometry (n) were estimated through non-linear regression of the experimental data using a model for one binding site implemented in Origin (Origin 7.0, OriginLab). The dissociation constant (*K*_d_), the Gibbs energy change (Δ*G*), and the entropy change (–*T*Δ*S*) were obtained from basic thermodynamic relationships.

### 2.6 Crystallization, data collection and model resolution of *Ba*AAO complexed with 4-methoxybenzoic acid

Crystal generation of *Ba*AAO:4-methoxybenzoic acid complex was performed using 96-well plates and two commercial screening kits: JCSG plus (Molecular Dimensions) and JBscreen Basic (Jena Bioscience). A solution of 6 mg/mL protein in 0.1 mM sodium phosphate at pH 6.5 was pre-incubated with 0.5 mM 4-methoxybenzoic acid (solubilized in EtOH). The mixture was left on ice for 5 min. Typically, 0.6 µL of this mixture was added to 0.6 µL of mother liquor, and all sitting drops were equilibrated against 60 µL of the corresponding reservoir liquid at 18 °C. Optimal crystals were observed under condition A2 of the BASIC screen (12% *v*/*v* glycerol, 1.5 M ammonium sulfate, 0.1 mM Tris/HCl, pH 8.5). Crystals intended for data collection were soaked in a cryoprotectant solution with 20% (*v*/*v*) glycerol and rapidly cooled to 100 K with liquid nitrogen.

Diffraction data were collected using a Dectris Pilatus 6M detector and synchrotron radiation at the BL13-XALOC beamline of ALBA, Barcelona (Spain). The diffraction data sets were processed and scaled using XDS and SCALA from CCP4i (Kabsch, 1988; Collaborative Computational Project and Number 4, 1994). The structure of *Ba*AAO complex was determined and refined via Molrep from CCP4i (Vagin and Teplyakov, 1997), employing the structure of the *P. eryngii* complex (PDBid: 5CO1) as a search model. Automatic model building was conducted with REFMAC5 from CCP4i (Murshudov et al., 1997). Coot (Emsley and Cowtan, 2004) was employed for manual refinement, cofactor, and ligands addition, and Molprobity (Williams et al., 2018) was used to evaluate the model quality. Atomic coordinates have been deposited in the Protein Data Bank (PDB) with accession code 9AVH. Pairwise Structure Alignment was performed using TM-align algorithm, sensitive to global topology (Zhang and Skolnick, 2005). Cavities and structural accessibility were analyzed using HOLLOW server (Ho and Gruswitz, 2008), and PyMOL (Delano, 2002) was used to generate the structural superpositions and all structural figures. Docking models of AAO:BQ complexes were constructed manually using the editing mode of the PyMOL (Delano, 2002) with *Pe*AAO (5OC1) and *Ba*AAO (9AVH) structures and BQ (PLQ).

## 3 Results and discussion

### 3.1 Influence of the electron acceptor on the catalytic mechanism and redox state during turnover

Bi-substrate kinetics of *Ba*AAO and *Pe*AAO were assayed for the oxidation of 3-Cl-4-methoxybenzyl alcohol using either O_2_ or BQ as electron acceptors. These kinetics were compared with those recently published with 4-methoxybenzyl alcohol (Ferreira et al., 2023). The Hanes-Woolf primary plot ([A]/*v*_0_ vs. [A]) for both acceptors resulted in straight lines with intersection points on the *y*-axis, indicating that the reactions follow a ping-pong mechanism (Supplementary Figure S1). Therefore, the steady-state kinetic parameters were determined by fitting the experimental data to Eq. 2, which describes a ping-pong mechanism (Table 1).

**TABLE 1:** Steady-state kinetic parameters of AAOs for 4-methoxybenzyl and 3-Cl-4-methoxybenzyl alcohols substrates with O_2_ or BQ as electron acceptors in 50 mM phosphate, pH 6.0 at 25 °C.

Alcohol substrate	Enzyme	e^−^ acceptor	*k*_cat_ (s^−1^)	*K*_m(alcohol)_ (µM)	*K*_m(acceptor)_ (µM)	*k*_cat_/*K*_m(alcohol)_ (s^−1^mM^−1^)	*k*_cat_/*K*_m(acceptor)_ (s^−1^mM^−1^)
4-Methoxybenzyl 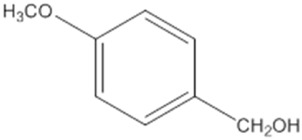	*Pe*AAO^a^	O_2_	201 ± 3	50 ± 2	176 ± 1	4,022 ± 135	1,139 ± 35
		BQ	49 ± 4	7 ± 1	48 ± 6	6,590 ± 963	1,025 ± 154
	*Ba*AAO^a^	O_2_	74 ± 1	331 ± 13	134 ± 5	222 ± 9	549 ± 23
		BQ	31 ± 3	82 ± 10	70 ± 9	381 ± 58	450 ± 70
3-Cl-4-methoxybenzyl 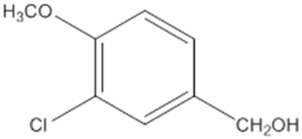	*Pe*AAO	O_2_	43 ± 1	11 ± 1	67 ± 2	3,806 ± 122	647 ± 22
		BQ	21 ± 1	4 ± 1	29 ± 2	5,874 ± 481	722 ± 64
	*Ba*AAO	O_2_	70 ± 1	94 ± 4	225 ± 8	747 ± 32	312 ± 12
		BQ	23 ± 1	26 ± 4	66 ± 12	910 ± 146	353 ± 65

^a^ Data from Ferreira et al. (2023).

Contrary to what has been reported for the oxidation of 4-methoxybenzyl alcohol, *Ba*AAO exhibited turnovers 2-fold higher than *Pe*AAO for the oxidation of 3-Cl-4-methoxybenzyl alcohol using O_2_ as electron acceptor. Nevertheless, *Ba*AAO and *Pe*AAO showed similar *k*_cat_ values when comparing alcohol oxidation using BQ as the electron acceptor. Additionally, the lower Michaelis-Menten constant value for 3-Cl-4-methoxybenzyl alcohol (*K*_m(alcohol)_) makes *Pe*AAO more efficient than *Ba*AAO at oxidizing this substrate. Furthermore, *Ba*AAO was less efficient for the oxidation of alcohol substrates acting either as oxidase (up to 18- and 17-fold lower *k*_cat_/*K*_m(alcohol)_, respectively) or as dehydrogenase (5 to 6-fold lower) than *Pe*AAO, which showed similar catalytic efficiency for the oxidation of both alcohol substrates. On the contrary, *Ba*AAO was more efficient at oxidizing 3-Cl-4-methoxybenzyl alcohol than 4-methoxybenzyl alcohol because of its higher affinity for this substrate.

Regarding the nature of the acceptor on catalysis, both enzymes exhibited higher affinity and lower turnovers for BQ than for O_2_. This was translated into similar catalytic efficiencies for the reduction of both acceptors, with *Pe*AAO being the most efficient. Furthermore, the affinity of both proteins for O_2_ varies depending on alcohol substrate similarly to that previously reported (Ferreira et al., 2015), while the affinity for BQ resulted in quite similar values. Additionally, BQ considerably improved the affinity for alcohol substrates [up to 7-fold lower *K*_m(alcohol)_] with both enzymes being slightly more efficient as dehydrogenases than oxidases for alcohol oxidation. A similar enhancement in catalytic efficiency using quinone as electron acceptors was previously reported for other GMC oxidases associated with lignocellulose decomposition, such as GOX and P2O (Leitner et al., 2001; Leskovac et al., 2005; Herzog et al., 2019).

**FIGURE 1:**
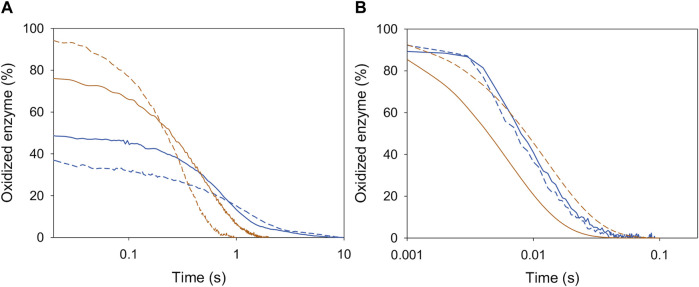
Redox state during turnover of *Pe*AAO (orange lines) and *Ba*AAO (blue lines) with O_2_ **(A)** and BQ **(B)** as electron acceptors. Enzymes (∼10 µM) were mixed with saturating concentrations of 4-methoxybenzyl alcohol (solid lines) or 3-Cl-4-methoxybenzyl alcohol (dashed lines) in 50 mM sodium phosphate, pH 6.0 at 25 °C. The lines depict the time course of absorbance changes at the maxima of the flavin band-I (462 nm).

An analysis of the redox state of *Ba*AAO and *Pe*AAO cofactors during steady-state turnover was conducted to gain insight into their rate-limiting steps during oxidation of 4-methoxybenzyl or 3-Cl-4-methoxybenzyl alcohols, acting both as dehydrogenases and oxidases. For this purpose, spectral changes of *Ba*AAO and *Pe*AAO were recorded after mixing with a saturating concentration of alcohol under air atmosphere or in the presence of BQ (under anaerobic conditions). During turnover conditions, as depicted in the lag phase in Figure 1, enzymes undergo cycling, and the absorbance of flavin band I provides information about their redox state forms. Moreover, the extent of these lag phases reflects the relative rates of protein reduction by each alcohol compared to their oxidation by O_2_ or BQ.

In the case of BQ acting as the electron acceptor, *Pe*AAO was predominantly in the oxidized state (85%–92%) during its turnover (Figure 1B), regardless of the alcohol substrate used. This aligns with results using O_2_ as electron acceptor (Ferreira et al., 2009; Ferreira et al., 2015) (Figure 1A). These findings indicate that, for *Pe*AAO, whether acting as an oxidase or dehydrogenase, its oxidative half-reaction is significantly faster that the reductive one. In contrast, the redox state of *Ba*AAO during turnover depends on the electron acceptor used. The enzyme remains predominantly oxidized (∼90%) when BQ is the electron acceptor with both alcohols tested (Figure 1B), while an increase in the reduced form of *Ba*AAO (up to 50% and 60% for 4-methoxybenzyl and 3-Cl-4-methoxybenzyl alcohols, respectively) is observed in the presence of O_2_. This suggests that the rates for the reductive and oxidative half-reactions might be balanced (Figure 1A). Moreover, turnover experiments suggest that both AAOs are more efficient acting as dehydrogenases than oxidases, in agreement with the catalytic efficiencies reported with 4-methoxybenzyl or 3-Cl-4-methoxybenzyl alcohols (Table 1).

### 3.2 Rapid kinetics of the two half-reactions

In the light of the steady-state results, the reductive and oxidative half-reactions of *Ba*AAO and *Pe*AAO were scrutinized to provide a detailed description of their catalytic mechanism during oxidation of 4-methoxybenzyl alcohol and 3-Cl-4-methoxybenzyl alcohols with both O_2_ and BQ as electron acceptors.

**FIGURE 2:**
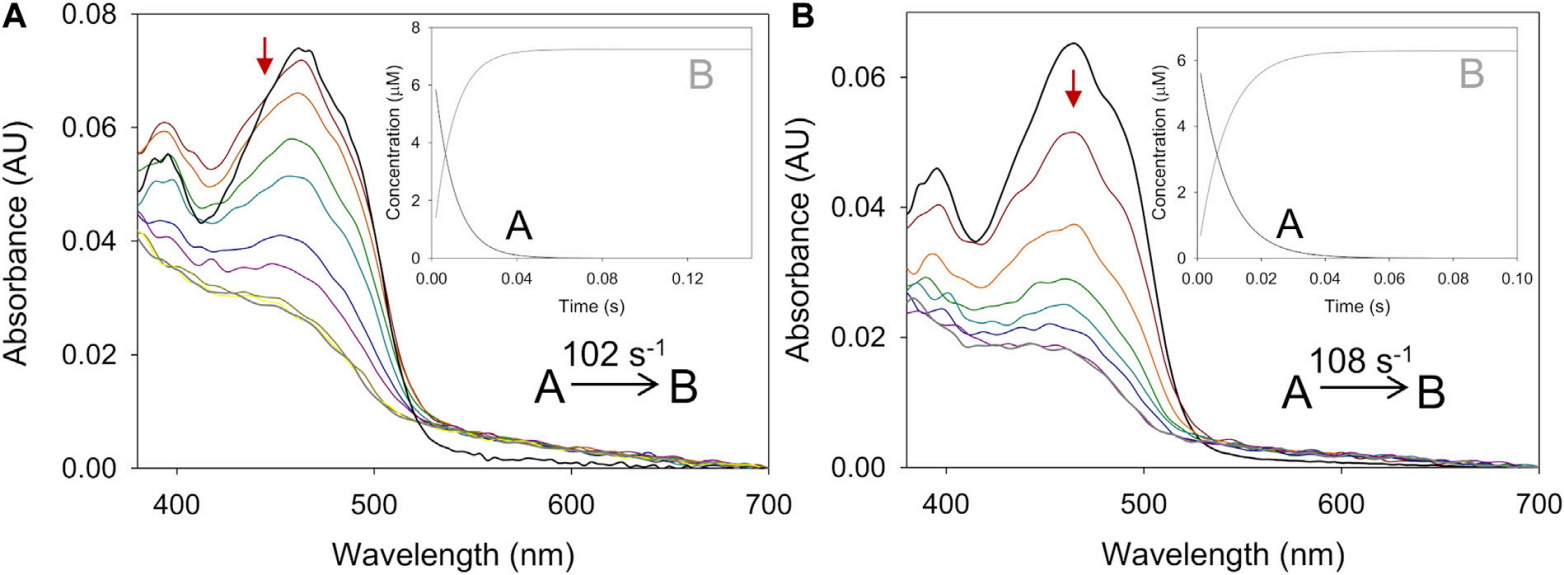
Time course of the reduction of *Ba*AAO with 4-methoxybenzyl and 3-Cl-4-methoxybenzyl alcohols. **(A)** Spectra of *Ba*AAO (∼10 µM) mixed with 4-methoxybenzyl alcohol (5 mM) measured at 0.003, 0.004, 0.006, 0.008, 0.014, 0.02, 0.05, 0.1, and 0.15 s after mixing. **(B)** Spectra of *Ba*AAO (∼10 µM) mixed with 3-Cl-4-methoxybenzyl alcohol (3 mM) measured at 0.003, 0.005, 0.01, 0.015, 0.02, 0.025, 0.06, and 0.1 s after mixing. Black lines correspond to the oxidized enzyme before mixing and gray lines correspond to the last spectrum after total reduction. The red arrows indicate the direction of spectral change observed when the time is increased. Reactions were performed in 50 mM sodium phosphate, pH 6.0 at 25 °C. Insets show evolution of species A (black line) and B (gray line) after data fitting to a one-step process.

In the reductive half-reaction, the spectral changes observed for *Ba*AAO reduction by both alcohols fitted well to a one-step model (A → B), consistent with the two-electron reduction of the flavin (Figure 2). The values of the rate constants (*k*_obs_) at different substrate concentrations exhibited a hyperbolic dependence on the alcohol concentration in both cases (Supplementary Figure S2). Fitting the experimental values to Eq. 3 allowed the determination of the reduction rate constant (*k*_red_) for each alcohol and their dissociation constant [*K*_d(alcohol)_]. No reverse reaction was detected for any of the alcohol substrates with *k*_rev_ values close to zero. A similar irreversible hydride transfer process was previously reported for *Pe*AAO (Ferreira et al., 2009; Ferreira et al., 2015).

The reduction rates for *Ba*AAO with both substrates (Table 2) were higher than the corresponding turnovers in the presence of either O_2_ or BQ (1.7-fold and 3–5-fold higher, respectively). This indicates that flavin reduction is far from being the rate-limiting step in catalysis and that the reduction is not affected by the presence of chlorine substituent in 3-Cl-4-methoxybenzyl alcohol. Nevertheless, it must be noted that the *K*_d_ value for 4-methoxybenzyl alcohol is higher than for 3-Cl-4-methoxybenzyl alcohol, which also agrees with their estimated *K*_m_. The same difference among turnovers and *k*_red_ also applies to *Pe*AAO with 3-Cl-4-methoxybenzyl alcohol using O_2_ and BQ (with *k*_red_ 2-, 4-fold higher than *k*_cat,_ respectively), and with 4-methoxybenzyl using BQ (with *k*_red_ 5-fold higher than *k*_cat_). *K*_d_ for 3-Cl-4-methoxybenzyl alcohol is lower (higher affinity) than for 4-methoxybenzyl alcohol in *Pe*AAO, what leads us to think that the accommodation of the chlorinated substrate or the release of the aldehyde product may affect *k*_cat_ (4-fold lower than for the other substrate). However, the reductive half-reaction with 4-methoxybenzyl alcohol must be the rate-limiting step in *Pe*AAO catalysis when acting as an oxidase.

**TABLE 2:** Transient-state kinetic constants for the reductive and oxidative half reactions of AAOs with 4-methoxybenzyl and 3-Cl-4-methoxybenzyl alcohols. Reduction experiments were performed at 25 °C under anaerobic conditions in 50 mM sodium phosphate, pH 6.0. Reoxidation assays for both enzymes were performed at 12 °C.

Alcohol substrate	Enzyme	*k*_red_ (s^−1^)	*K*_d(alcohol)_ (µM)	*k*_red_/*K*_d(alcohol)_ (s^−1^mM^-1^)	*k*_ox(O_2_)_ (s^−1^mM^−1^)	*k*_ox(BQ)_ (s^−1^mM^−1^)
4-Methoxybenzyl	*Pe*AAO	251 ± 16	80 ± 13	3,138 ± 557	770 ± 40^a^	792 ± 52
	*Ba*AAO	117 ± 2	765 ± 47	153 ± 10	216 ± 13	175 ± 12
3-Cl-4-methoxybenzyl	*Pe*AAO	96 ± 3	21 ± 3	4,486 ± 725	790 ± 20^b^	760 ± 104
	*Ba*AAO	121 ± 11	300 ± 91	404 ± 127	123 ± 16	84 ± 9

^a^ Data from Carro et al. (2018a).

^b^ Data from Ferreira et al. (2015).

**FIGURE 3:**
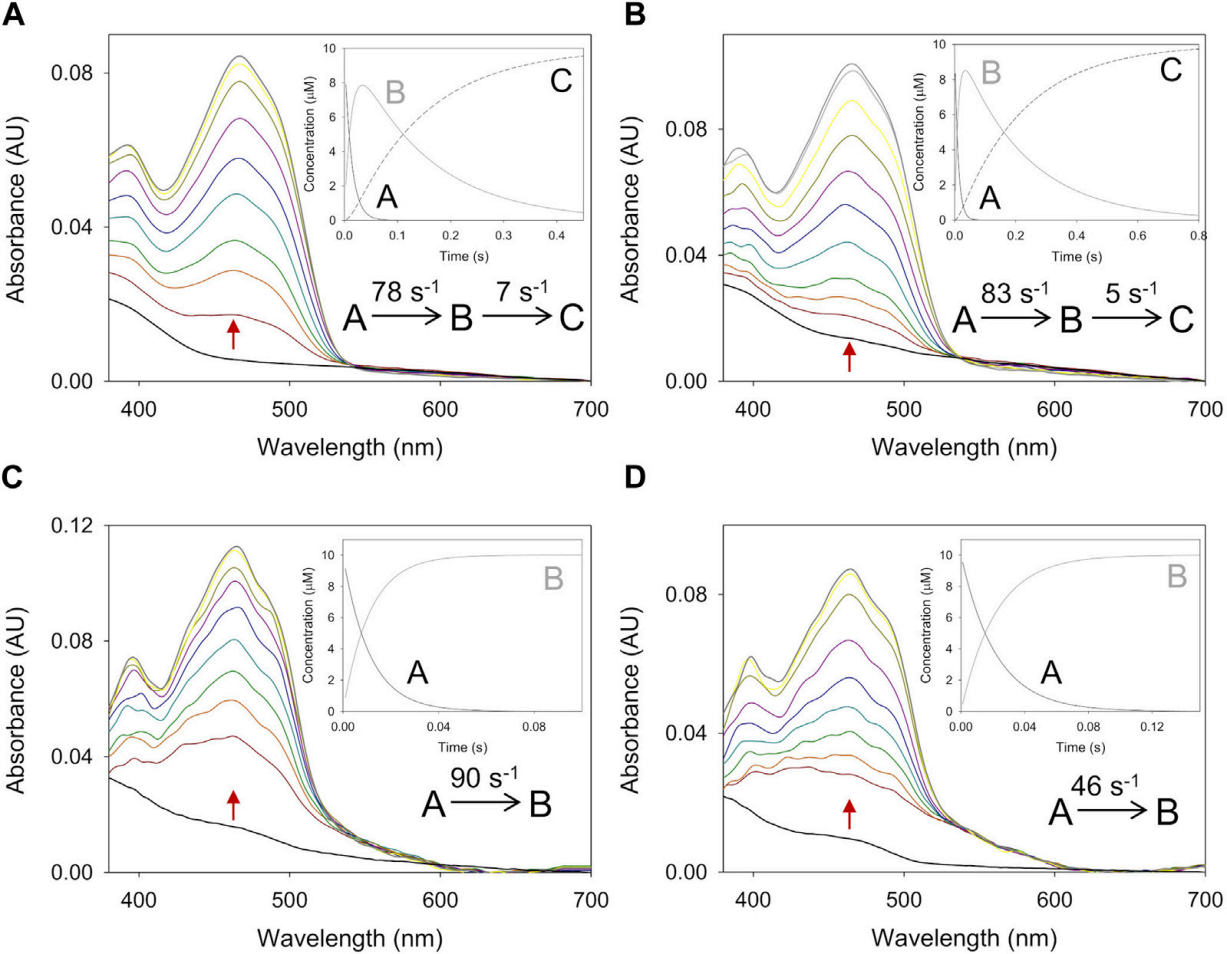
Time course of the reoxidation of *Pe*AAO and *Ba*AAO with BQ. **(A)** Spectra of *Pe*AAO (∼10 µM) reduced by 4-methoxybenzyl alcohol and then measured at 0.007, 0.014, 0.021, 0.042, 0.084, 0.154, 0.252, 0.35, and 0.49 s after mixing with BQ (50 µM). **(B)** Spectra of *Pe*AAO (∼10 µM) reduced by 3-Cl-4-methoxybenzyl alcohol and then measured at 0.002, 0.006, 0.01, 0.02, 0.04, 0.1, 0.2, 0.3, 0.6, and 0.8 s after mixing with BQ (50 µM). **(C)** Spectra of *Ba*AAO (∼10 µM) reduced by 4-methoxybenzyl alcohol and measured at 0.001, 0.005, 0.007, 0.010, 0.015, 0.02, 0.025, 0.05, and 0.1 s after mixing with BQ (400 µM). **(D)** Spectra of *Ba*AAO (∼10 µM) reduced by 3-Cl-4-methoxybenzyl alcohol measured at 0.001, 0.004, 0.006, 0.01, 0.015, 0.025, 0.05, 0.1, and 0.15 s after mixing with BQ (350 µM). Black lines correspond to the reduced enzyme by the alcohol before mixing with BQ and gray lines correspond to the last spectrum after total reoxidation. The red arrows indicate the direction of spectral change observed when the time is increased. Reactions were performed in 50 mM sodium phosphate, pH 6.0 at 12 °C. Insets show evolution of species A (black line), B (gray line) and C (dashed black line) after data fitting to a one-step or two-step process.

Similarly, the oxidative half-reaction was measured by following flavin reoxidation with O_2_ or BQ after protein reduction by either 4-methoxybenzyl or 3-Cl-4-methoxybenzyl alcohols. In all cases, the spectral changes indicated a two-electron oxidation of flavin hydroquinone form (FADH^−^) to flavin quinone (FAD^+^) (Figure 3; Supplementary Figure S3) as previously reported for *Pe*AAO with O_2_ (Hernández-Ortega et al., 2012). For *Ba*AAO, these spectral changes fitted well a one-step model (A → B) with both electron acceptors. This *k*_obs A→B_ exhibited linear dependence on the concentration of electron acceptor, allowing the determination of a second-order rate constant ^app^ *k*_ox_, after fitting to Eq. 4 (Supplementary Figure S4). The estimated *k*_ox_ indicated that *Ba*AAO reoxidizes slightly faster in the presence of O_2_. Similarly to what happens with *Pe*AAO, the reoxidation of *Ba*AAO may occur through a stepwise reaction involving two separated kinetic steps that are not spectroscopically distinguishable, during which one proton from the solvent and one electron and hydrogen atom from the FAD moiety are transferred to O_2_ yielding H_2_O_2_ (Hernández-Ortega et al., 2012).

The spectral reoxidation changes for *Pe*AAO in the presence of BQ fitted well a one-step model with the exception of the first two lowest concentrations that fitted a two-step model (A → B → C) describing a biphasic pattern (Figure 3). The first phase accounts for more than 50% and 85% of the total amplitude for 50 and 100 µM of BQ, respectively. The *k*_ox A→B_ showed linear dependence on BQ concentration, being the estimated bi-molecular rate constants similar to those with O_2_. Regarding the second phase (*k*_ox B→C_), it was dependent on BQ’s concentration and in the range of 5–10 and 15–20 s^−1^ when reoxidized with 50 and 100 µM of BQ, respectively.

For both enzymes, the flavin reoxidation constants were similar for the two alcohols used as flavin reductant and several orders of magnitude larger than those for reoxidation of free reduced flavins (Mattevi, 2006). This indicates that enzymes activate the reduced flavin for reaction with both electron acceptors. When comparing the reoxidation rates between enzymes, *Pe*AAO is up to 6-fold and 9-fold more efficient at reducing O_2_ and BQ, respectively than *Ba*AAO. For both enzymes, a reverse rate constant (*k*_rev_), corresponding to the intercept of the *y*-axis, was observed with values in the range of ∼14–19 s^−1^ for *Ba*AAO with both acceptors and of 17 s^−1^ and 55 s^−1^ for *Pe*AAO with O_2_ and BQ, respectively.

### 3.3 The interaction of *Ba*AAO and *Pe*AAO with the products of their catalytic reactions

**FIGURE 4:**
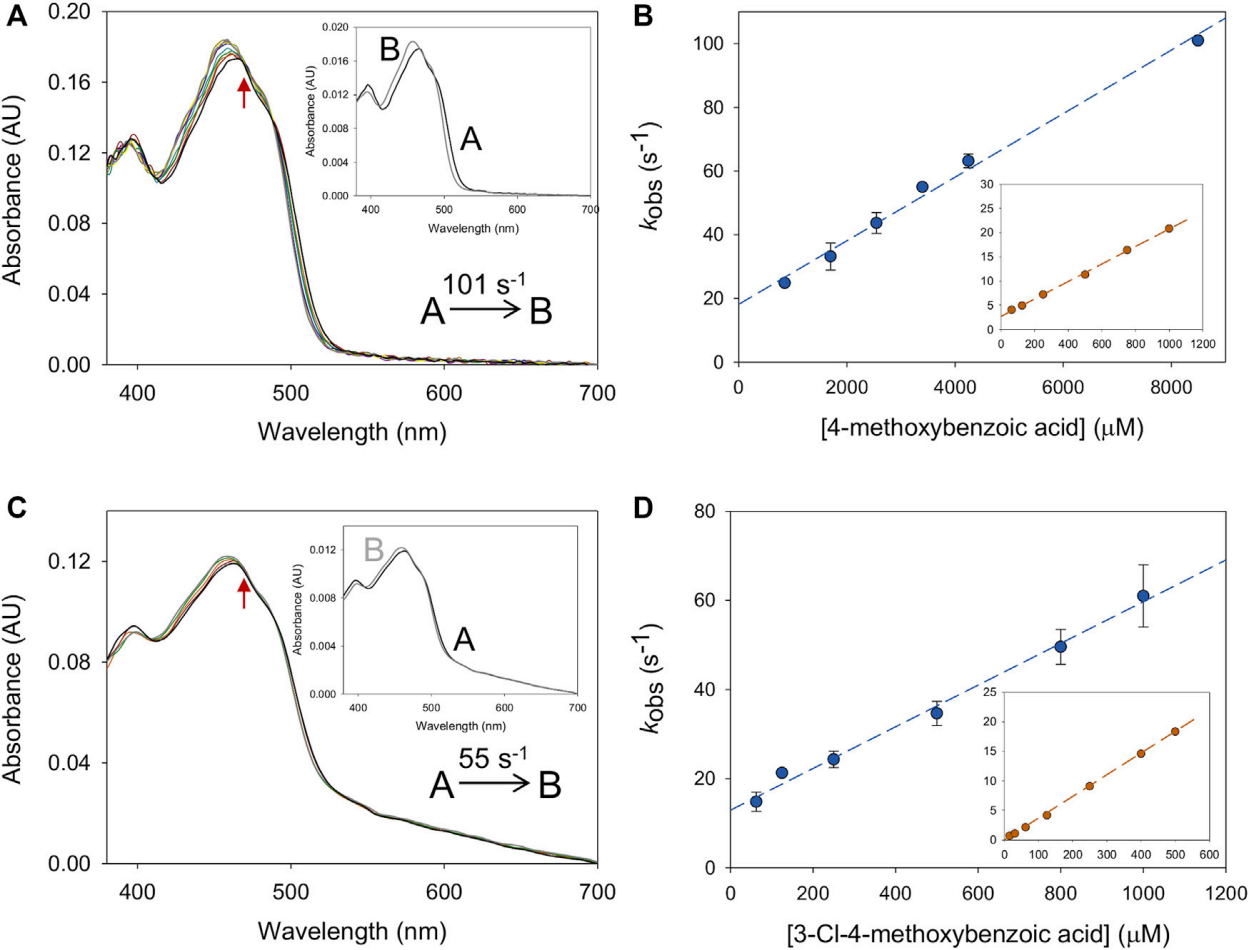
Formation of *Ba*AAO: acid complexes. **(A)** The spectral changes for *Ba*AAO (∼10 µM) mixed with 4-methoxybenzoic acid (8 mM) measured at 0.001, 0.003, 0.006, 0.008, 0.01, 0.02, 0.03, 0.04, 0.05, and 0.1 s after mixing. Inset shows the evolution of species A into species B. **(B)** Plots of the *k*_obs_ obtained for *Ba*AAO at increasing concentrations of acid. The inset shows the fittings for *Pe*AAO (Carro et al., 2018a). **(C)** The spectral changes for *Ba*AAO (∼10 µM) mixed with 3-Cl-4-methoxybenzoic acid (0.8 mM) measured at 0.003, 0.006, 0.021, 0.051, and 0.102 s after mixing. The red arrows indicate the direction of spectral change observed when the time is increased. Inset shows the evolution of species A (black line) into species B (gray line). **(D)** Plots of the *k*_obs_ obtained for *Ba*AAO at increasing concentration of 3-Cl-4-methoxybenzoic acid. The inset shows the fittings for *Pe*AAO. Reactions were performed in 50 mM sodium phosphate, pH 6.0 at 12 °C.

The differences observed between the *k*_cat_ and *k*_red_ values for *Ba*AAO and *Pe*AAO reduction by either 4-methoxybenzyl and 3-Cl-4-methoxybenzyl alcohols led us to investigate whether product release could have some effect on turnover. The BQ absorption in the flavin bands, however, prevented titration assays for monitoring the flavin spectral perturbation upon protein:BQ complex formation. Additionally, the formation of the enzyme:aldehyde complex was so fast that it cannot be detected by stopped flow spectrometry. Therefore, the kinetics of enzyme:product complex formation and dissociation were performed with 4-methoxybenzoic and 3-Cl-4-methoxybezoic acids, the final products after aldehyde oxidation. The spectral changes observed for the complex formation showed a slight shift in the absorption maximum and in the intensity of flavin bands, which were fitted well to a one-step model (A → B) (Figures 4A, C). The *k*_obs_ exhibited linear dependence on the concentration of the corresponding benzoic acids fitting to Eq. 5 (Figures 4B, D). This allowed obtaining the second order constant for complex formation (*k*_on_) and the rate of dissociation (*k*_off_) shown in Table 3. The calculated *k*_on_ values suggested that the diffusion of the 4-methoxybenzoic in the active site of both enzymes was slower than that of 3-Cl-4-methoxybezoic acid (approximately 2-fold and 5-fold for *Pe*AAO and *Ba*AAO, respectively). However, the *k*_off_ values for *Ba*AAO with both acids were similar and higher than those for *Pe*AAO. Notably, no dissociation of 3-Cl-4-methoxybenzoic acid from the active site of *Pe*AAO was observed under assay conditions, suggesting a very slow diffusion rate of the reaction product derived from the oxidation of 3-Cl-4-methoxybezyl alcohol. Altogether, these results indicate a faster release of the acids from the active site in *Ba*AAO compared to *Pe*AAO. Additionally, the determined *K*_d(acid)_ values (*K*_d_ = *k*_off_/*k*_on_) for *Ba*AAO were significantly higher than that for *Pe*AAO, suggesting a weaker interaction. As a whole, these observations are consistent with the higher *K*_m_ values obtained for the oxidation of alcohols by *Ba*AAO compared to those of *Pe*AAO.

**TABLE 3:** Formation and dissociation rates of AAO:4-methoxybenzoic acid or AAO:3-Cl-4-methoxybenzoic acid complexes. Experiments were performed in 50 mM sodium phosphate, pH 6.0 at 12 °C.

Ligand	Enzyme	*k*_on_ (s^−1^mM^-1^)	*k*_off_ (s^−1^)	*K*_d(acid)_ (µM)
4-Methoxybenzoic acid	*Pe*AAO^a^	18 ± 1	3 ± 1	149 ± 10
	*Ba*AAO	10 ± 1	18 ± 2	1815 ± 197
3-Cl-4-methoxybenzoic acid	*Pe*AAO	37 ± 1	nd	nd
	*Ba*AAO	47 ± 2	13 ± 1	276 ± 38

^a^ Data from Carro et al. (2018a).

Nd: not detected.

Additionally, the thermodynamics of the interaction with BQ were evaluated by ITC (Table 4; Supplementary Figure S5). The binding isotherms fitted well a model considering a single binding site that revealed a much higher affinity for BQ in the case of *Ba*AAO than for *Pe*AAO (*K*_d_ values of 0.05 µM and 27.1 µM, respectively). Ligand binding for both enzymes was driven by a similar and large favorable enthalpic contribution, while the entropic contribution was unfavorable. Because the interaction was measured in Tris buffer, which has a considerable ionization enthalpy, the intrinsic enthalpies and entropies might be somewhat different to the observed parameters. However, given the large values determined, it may be argued that similar thermodynamic signatures (enthalpically-driven binding) will result after removing the buffer contribution, if any. This common thermodynamic profile is indicative of specific forces contributing to the interaction within the active site of both enzymes. Moreover, the calculated free energies indicate a more favorable interaction of BQ with *Ba*AAO than with *Pe*AAO (−9.9 kcal/mol and −6.2 kcal/mol, respectively).

**TABLE 4:** Thermodynamic parameters determined for the interaction of AAOs with BQ.

Ligand	Enzyme	*K*_*d*_(BQ) (µM)	Δ*H* (kcal/mol)	–TΔ*S* (kcal/mol)	Δ*G* (kcal/mol)
BQ	*Pe*AAO	27.1	−25.1	18.9	−6.2
	*Ba*AAO	0.05	−21.4	11.5	−9.9

ITC assays were performed at 25 °C in 50 mM Tris/HCl, pH 7.0. The thermodynamic parameters were calculated using well-known relationships: *K*_d_ = (*K*_a_)^−1^, Δ*G* = *RT*, ln*K*_d_ and–*T*Δ*S* = Δ*G*–Δ*H*., Errors considered in the measured parameters (±30% for *K*_d_, ±0.4 kcal/mol for Δ*H*, and–TΔ*S*, and ±0.1 kcal/mol for Δ*G*) were considered larger than the standard deviation between replicates and the numerical error after the fitting analysis).

### 3.4 Crystallographic structure of *Ba*AAO:4-methoxybenzoic acid complex

The crystal structure of *Ba*AAO in complex with 4-methoxybenzoic acid was solved at a resolution of 1.8 Å in the space group P212121. The asymmetric unit contains the protein molecule, one FAD molecule, one 4-methoxybenzoic acid, six sulfate ions, five glycerol molecules, and 377 water molecules. The structure of the homologous complex from *P. eryngii* (PDBid: 5OC1, with 47% sequence identity) served as a template for molecular replacement. Data collection and refinement statistics are summarized in Table 5. In the comparison of *Pe*AAO and *Ba*AAO complex structures, an RMSD of 1.35 Å for 558 Cα highlights their overall folding similarity. Additionally, among PDB structures, *Ba*AAO shares also substantial similarity with the pyranose dehydrogenase from *Agaricus meleagris* (PDBid: 4H7U) (Supplementary Table S1) and a recently reported AAO from *Thermothelomyces thermophilus*—synonym *Myceliophthora thermophila*—(PDBid: 6O9N) (Kadowaki et al., 2020), with 37% and 28% sequence identities and values for RMSD of 1.91 and of 2.9 Å for 543 Cα, respectively.

**TABLE 5:** Data collection and structural refinement statistics of *Ba*AAO:4-methoxybenzoic acid.

Data collection statistics	
Space group	P2_1_2_1_2_1_
Unit cell parameters	
a, Å	56.04
b, Å	82.75
c, Å	116.84
Wavelength, Å	0.979257
Resolution, Å	116.84–1.80 (1.90–1.80)
N^o^ of unique reflections	50514 (7,207)
Redundancy	6.7 (6.8)
Completeness, %	98.9 (98.2)
Mn(I)/sd	12.2 (2.7)
R_merge_ ^a^	0.097 (0.843)
Refinement statistics	
Resolution range, Å	67.53–1.80
Protein non-hydrogen atoms	4,413
Ligand non-hydrogen atoms	124
Solvent non-hydrogen atoms	378
R_work_ (%)	14.57
R_free_ ^b^ (%)	18.76
rmsd bond lenght, Å	0.008
rmsd bond angles, °	1.695
Average B-factor, Å^2^	22.47

Values in parentheses correspond to the highest resolution shell.

^a^ R_sym_ = Σ| I - I_av_ |/Σ I, where the summation is over symmetry equivalent reflections.

^b^ R calculated for 5% of data excluded from the refinement.

**FIGURE 5:**
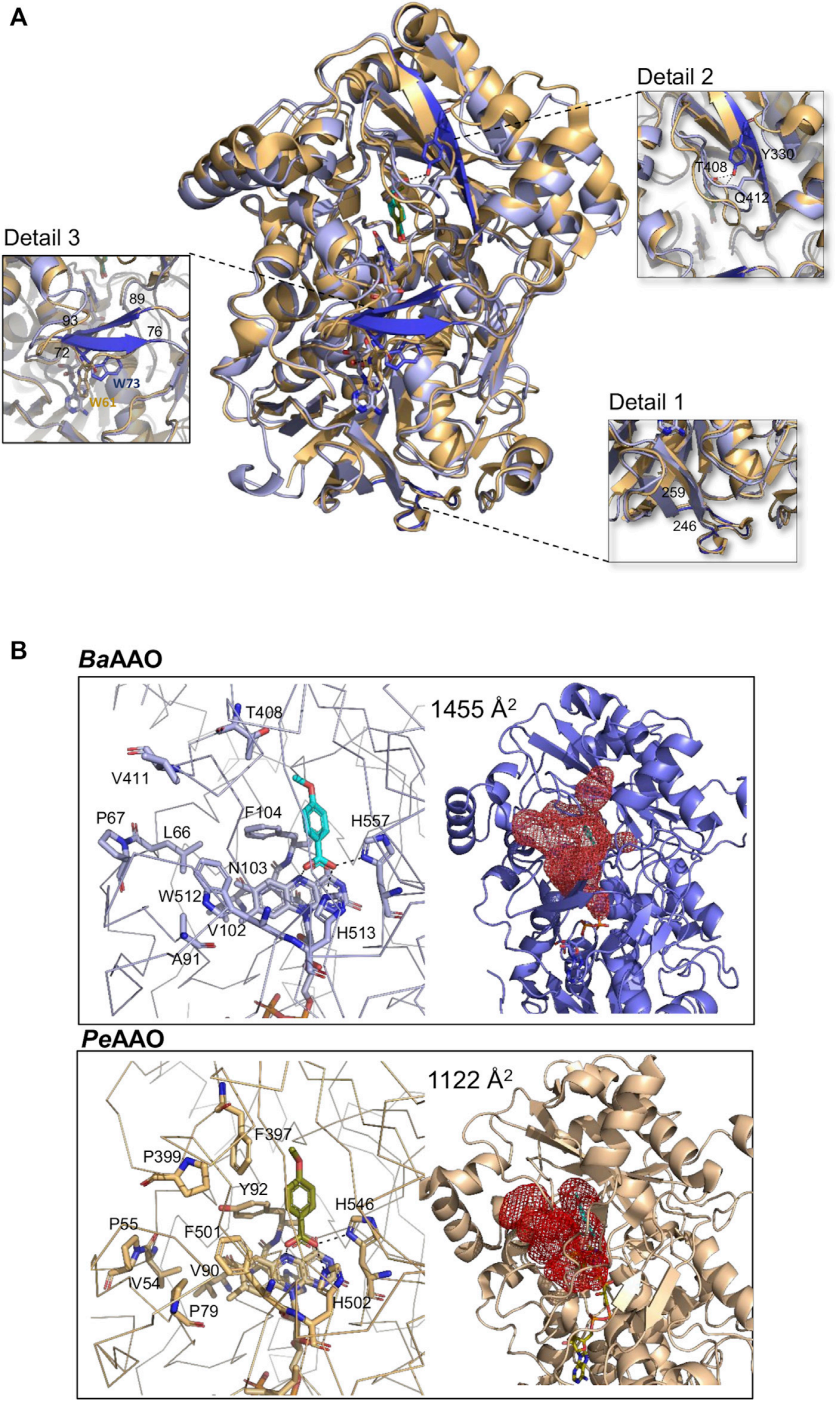
Crystallographic structure. **(A)** The structural superposition of *Ba*AAO:4-methoxybenzoic complex (9AVH, blue) on *Pe*AAO:acid complex (5OC1, light orange). Dark blue highlights some structural discrepancies between them, accompanied by detailed images of each difference in squares that are connected to the corresponding zone by dashed lines. **(B)** Substrate binding sites and access channels to the active sites in *Ba*AAO and *Pe*AAO complexes. Residues lining the pathway to the active site and key residues in the active site are shown in sticks with carbons in light blue and light orange, respectively. The 4-methoxybenzoic acids are displayed with cyan and olive-green carbons, respectively. The HOLLOW server was used to display the access channels (Ho and Gruswitz, 2008).

As a member of the GMC oxidoreductase superfamily, *Ba*AAO complex structure contains two domains: a highly conserved FAD-binding domain and a substrate-binding domain with 4-methoxybenzoic acid placement (Figure 5; Supplementary Figure S6A). Despite the similarity between *Ba*AAO and *Pe*AAO structures, detailed comparisons (Figure 5) reveal nuanced differences. Specifically, the FAD binding domain is similarly organized by a five-stranded parallel β-sheet, surrounded by three α-helices and linked to a three-stranded antiparallel β-sheet. However, unlike *Pe*AAO, this second β-sheet is not linked to two additional antiparallel strands. Instead, the corresponding fragment in *Ba*AAO (residues 246–259) forms a loop, similar to others GOX structures like that from *Aspergillus niger* (PDBid: 3QVR) (Figure 5A, detail 1). On the other hand, the substrate binding site of *Ba*AAO exhibits a six-stranded antiparallel β-sheet arranged similarly to that of *Pe*AAO. Notably, one of the strands in *Ba*AAO is twice as long, (comprising residues 325–333) compared to *Pe*AAO (319–322). This elongated strand in *Ba*AAO includes the Tyr330 residue, whose hydroxyl group is bound to the oxygen atoms of the Gln412 and Thr408 residues (Figure 5A, detail 2). These residues are located in a loop that is proposed to be part of the access channel for the substrate (Carro et al., 2018a). As a result, the tunnel in *Ba*AAO expands compared to *Pe*AAO. Additionally, a segment of the structure that links both domains, lacking specific secondary structure in *Pe*AAO, adopts a two-stranded β-sheet conformation in *Ba*AAO (residues 72–76 and 89–93, detail 3). In the *A. niger* GOX structure, these strands consist of only two residues each, noticeably shorter than those found in the *Ba*AAO structure (Kommoju et al., 2011).

In the active site, the cofactor FAD of *Ba*AAO complex adopts an elongated conformation, stabilized by polar contacts with residues in the N domain and several water molecules (Supplementary Figure S6B). The hydrogen-bonding network is similar to that of *Pe*AAO, except for the absence of interaction between the ribose part of FAD and a Trp residue. This residue is located in an interdomain segment that is unstructured in *Pe*AAO, but forms part of a strand in *Ba*AAO. In this complex, Trp73 (Trp61 in *Pe*AAO) is located at a distance and adopts an orientation that prevents any association with FAD (Figure 5A, detail 3).

Regarding the substrate binding site, in the *Ba*AAO complex, the carboxylic O atoms of the 4-methoxybenzoic acid are H bonded to the Nε atom of His513 (the catalytic base in *Pe*AAO), the Nδ atom of His557 (the other highly conserved His residue in GMC oxidoreductase superfamily), and the N5 atom of the FAD isoalloxazine ring (Figure 5B). Comparable interactions are also observed in the mentioned *Pe*AAO complex (Carro et al., 2017). Thus, *Ba*AAO substrate-like binding mode is compatible with alcohol oxidation by hydride transfer mechanism assisted by a catalytic base as previously described in other GMC proteins. Furthermore, Phe104, Trp512 and atoms of FAD create a hydrophobic environment for stabilization of some carbons of this product analogue. Finally, the substrate entrance at the active site of *Ba*AAO comprises residues Leu66, Pro67, Ala91, Val102, Asn103, Phe104, Thr408, and Trp512, identified through structural superimposition with the *Pe*AAO complex. Notably, the substrate channel in *Ba*AAO appears wider (Figure 5B) partially attributed to the presence of the Thr498 residue in its structure, rather than the Phe397 residue observed in *Pe*AAO, which leaves more space for the substrate to access. Moreover, eventually Phe104 and Trp512 should relocate to provide the substrate final access to the active site, akin to the proposal for Tyr92 and Phe501 in *Pe*AAO (Hernández-Ortega et al., 2011; Ferreira et al., 2015).

### 3.5 Overall AAO mechanism: dual activity as oxygen and quinone reductase and physiological relevance in lignin decay

**SCHEME 1:**
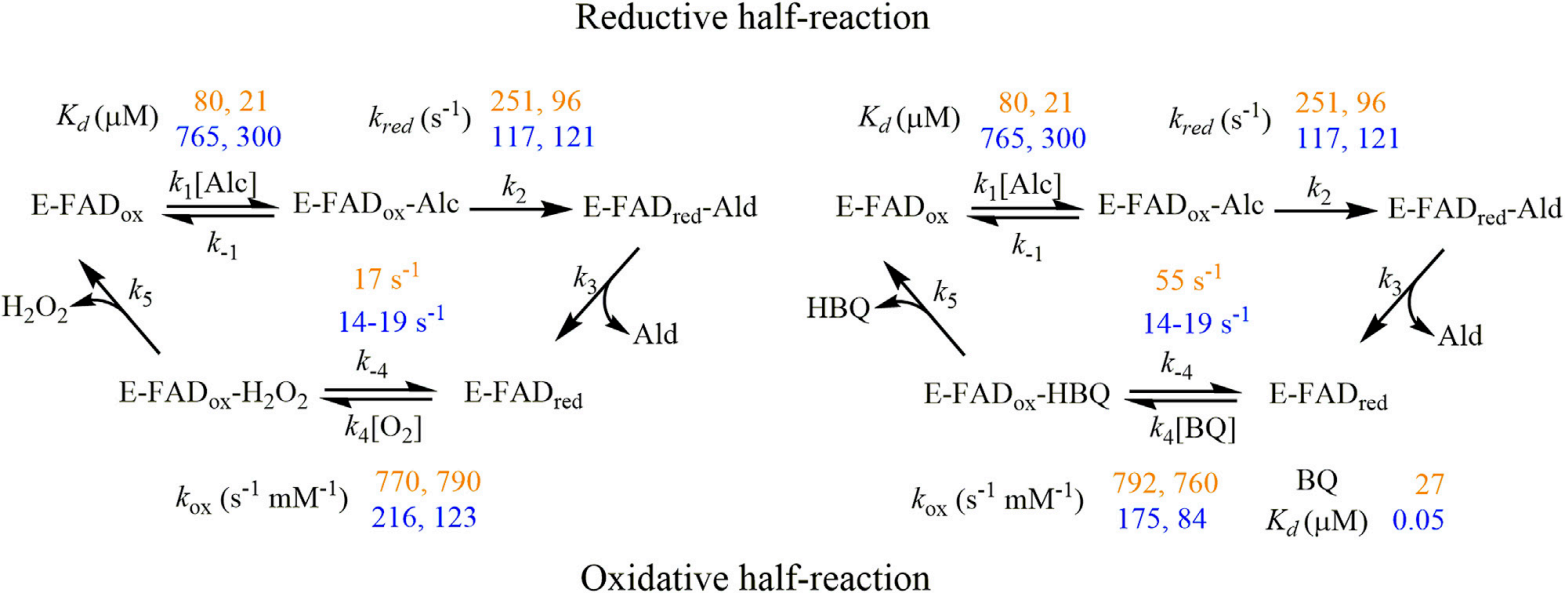
Proposed kinetic mechanism for AAO catalysis using oxygen (left) and BQ (right) as electron acceptors. The experimental kinetics parameters for *Pe*AAO and *Ba*AAO are written down in orange and yellow colors, respectively. The first and second values of each correspond to the assays performed with 4-methoxybenzyl and 3-Cl-4-methoxybenzyl alcohols, respectively.

In this work, we conducted a comparative analysis of the versatile oxidase and dehydrogenase activities of AAOs. Given the potential significance of this AAO’s dehydrogenase activity, we recently tackled its study in some extent using alternative electron acceptors such as the artificial redox dye 2,6-dichlorophenolindophenol, as well as benzoquinone (BQ) generated during lignin decay. Interestingly and contrary to what has been reported for *U. maydis* AAO (Couturier et al., 2016), the activity of several AAOs—including *Pe*AAO—with BQ was found to be similar to or even greater than that with oxygen when comparing catalytic efficiencies for the oxidation of 4-methoxybenzyl alcohol, a typical metabolite for *Pleurotus* species in their natural environments (Ferreira et al., 2023). This pointed toward a biological significance of AAO quinone reductase activity that led us to address this mechanistic study, involving *Pe*AAO and *Ba*AAO proteins in addition to 3-Cl-4-methoxybenzyl alcohol, a natural substrate of *Bjerkandera* AAO (de Jong et al., 1994). The global analysis of kinetic data obtained from different approaches reveals some interesting aspects required for the understanding of the quinone-reducing properties of this group of enzymes (Scheme 1). Both AAOs show lower turnover rates when BQ is used as electron acceptor than those with oxygen. Furthermore, these *k*_cat_ values, being markedly lower than the corresponding reduction constants (*k*_*red*_ up to 5 times higher for both enzymes), indicate that the flavin reduction is not the rate-determining step in the overall dehydrogenase reactions. Given the fact that oxidized proteins are the predominant state during turnover, the final step involved in hydrobenzoquinone release might be limiting the catalysis. Parameters for the titration of both proteins with BQ revealed stronger binding than those with alcohols (*K*_d_ values up to 26- and 15,000-fold higher for *Pe*AAO and *Ba*AAO, respectively) that may contribute to delaying the release of BQ from the active site. These results agree with previous studies conducted on GOX with quinones, suggesting that the diffusion of these compounds in and out of the active site may be the rate-determining step for the overall reaction, rather than the electron transfer between reduced protein and the acceptor (Mtemeri and Hickey, 2023). By contrast, when O_2_ was used as electron acceptor, up to 60% of *Ba*AAO is reduced during turnover, suggesting that the rates of flavin reoxidation influence the lower *k*_cat_ values compared to those of *k*_red_, limiting the reaction rate during catalysis with both alcohol substrates. In the case of the reaction of *Pe*AAO with 3-Cl-4-methoxybenzyl alcohol and O_2_, release of the aldehyde product may limit the catalysis. This is reinforced by the lack of diffusion of 3-Cl-4-methoxybenzoic acid from the protein active site compared to that reported for 4-methoxybenzoic acid. This agrees with previous simulations of ligand migration in *Pe*AAO, which indicate that the product release is limited due to both the reduced gated space and the need of displacement of the side chain of Phe397 to allow the product to exit (Carro et al., 2018a). In contrast, the expanded channel described for the *Ba*AAO structure agrees with the faster release of ligands from its active site compared to those observed for *Pe*AAO.

**FIGURE 6:**
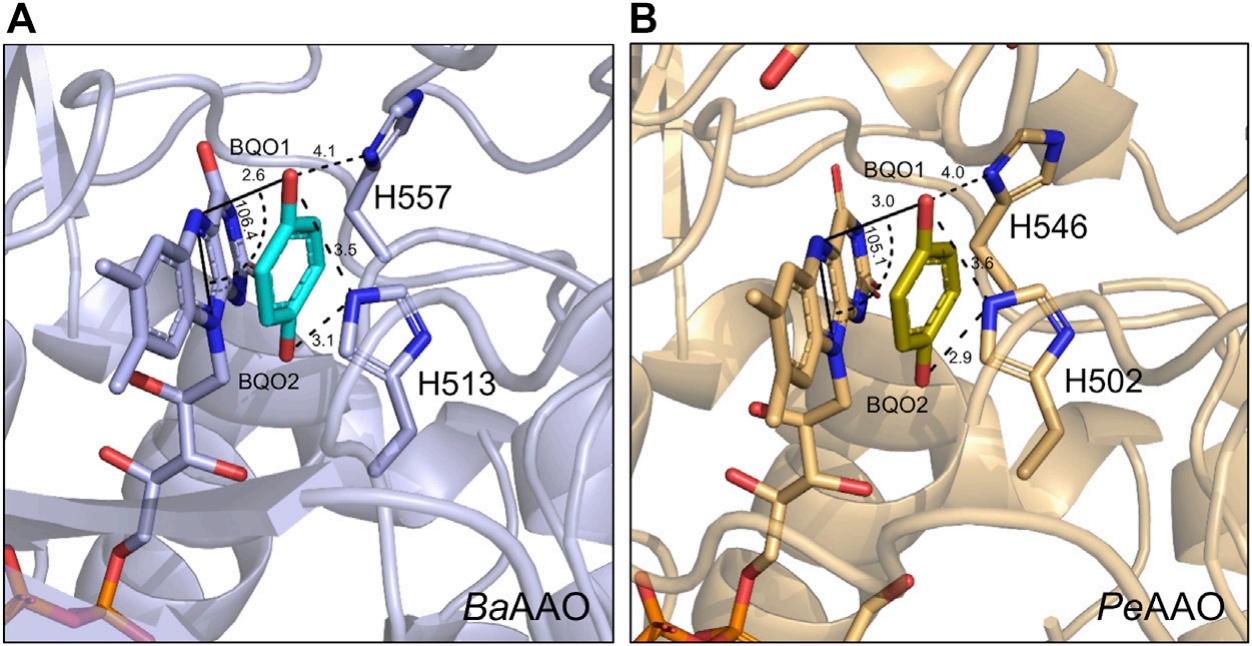
Docking models of **(A)** *Ba*AAO (9AVH, blue) and **(B)** *Pe*AAO (5OC1, light orange) with one BQ molecule (in cyan and olive-green C atoms, respectively) at their active site. FAD and the two catalytically relevant Histidine residues are shown in sticks. Models were edited with PyMOL. Relevant distances and angle are displayed with dashed lines with values in Å and degrees, respectively.

Regarding the oxidative-half-reaction, the hydroquinone FAD form of *Pe*AAO and *Ba*AAO was reoxidized with BQ as electron acceptor without the spectroscopic determination of any semiquinone intermediate. Furthermore, the high reoxidation rates for *Pe*AAO and *Ba*AAO were similar to those reported for GOX and do not suggest steric hindrances for BQ to get access to the FAD moiety (Mtemeri and Hickey, 2023). It is worth noting that the distance for electron transfer between the FAD and the redox site of the quinone molecules has been suggested to be a determining factor for the effectiveness of this electron transfer reaction (Leskovac et al., 2005; Tsuruoka et al., 2017). We performed some fruitless cocrystallization experiments with a high excess of BQ in the crystallization mixtures, which did not allow us to obtain the complex of AAO proteins with BQ. Thus, docking models were constructed to place a BQ molecule in the active-site pocket of these AAOs to elucidate its catalytically relevant position and the mechanism of reduction. In the *Ba*AAO model, all distances among the atoms of the isoalloxazine ring of FAD and BQ were kept in the range of 2.3–4.3 Å. In particular, the O1 of BQ, which accepts the hydride ion from N5 (FAD) during the reoxidation of this cofactor, is oriented to define an angle N10-N5-O1 of 106.4°, being at 2.6 Å distance from N5. The other O2 of BQ is 2.9 Å away from the Nɛ2 atom of His513. This geometry is consistent with conserved features of substrate positioning in flavoenzymes (Fraaije and Mattevi, 2000). The resulting position of BQ in both AAO:BQ complexes is compatible with a catalytic mechanism involving two-electron transfer from the FADH^−^ (hydroquinone form) to one of the oxygen of BQ, and the resulting hydrobenzoquinone anion could be neutralized by a protonated active site histidine [formed in previous reductive-half reaction (Hernández-Ortega et al., 2012)]. This proton transfer may require some reorientation of the quinone within the active site of AAO as was previously suggested for GOX (Figure 6) (Leskovac et al., 2005; Mtemeri and Hickey, 2023).

The quinone-reducing activity of AAO may play a relevant role in lignocellulose decomposition beyond its well-known role supplying H_2_O_2_ to fuel ligninolytic peroxidases and lytic polysaccharide monooxygenases (LPMOs) and in triggering Fenton reactions (Bissaro et al., 2017). Years ago, it was suggested that, in addition to oxygen, phenoxy radicals together with laccase/peroxidase-generated quinonoids could act as natural oxidizing substrates for AAOs during lignocellulose decay (Marzullo et al., 1995). Indeed, it has been reported that AAQOs phylogenetically related to AAO, reduce lignin-derived phenoxy radicals formed upon catalytic action of laccases and peroxidases, thereby preventing lignin repolymerization, as has been suggested for P2O (Ming-Qiang et al., 2014; Bugg, 2024).

The quinone and aromatic-radical reducing activity investigated in-depth in the present paper is an example of the involvement of AAOs in the quinone redox cycle that takes place during lignocellulose degradation (Sun et al., 2023). On the one hand, the hydroquinones generated drive the Fenton reactions, producing highly reactive oxidants during the non-enzymatic attack to polysaccharides during brown rot decomposition (Suzuki et al., 2006). Furthermore, hydroquinones may provide LPMOs with the electrons needed to cleave cellulose (Kracher et al., 2016). Therefore, AAOs and other GMC oxidoreductases can regenerate and recycle these redox mediators in order to activate LPMO for copper reduction and enzyme activation to cleave cellulose (Sützl et al., 2018).

On the other hand, hydroquinones produced by AAOs can serve as a natural redox mediators for the enzymatic attack to lignin carried out by laccases and peroxidases (Cañas and Camarero, 2010). Interestingly, the quinone-reducing activity of the GMC enzyme P2O participates with MnP in redox cycling of aromatic lignin model compounds (Herzog et al., 2019). Versatile oxidase and dehydrogenase activities encourage the precise coupling of AAO enzymes with the enzymatic arsenal of these white-rot fungi, promoting their efficient attack and lignocellulose-degrading capability that distinguishes them. Moreover, oxidoreductases with a broader specificity (promiscuous fuel spectrum) and the ability to utilize small redox-active molecules to act as electron mediators are emerging as promising candidates for bioelectrocatalysis applications such as enzymatic electrosynthesis and biofuels cells.

## Data Availability

The datasets presented in this study can be found in online repositories. The names of the repository/repositories and accession number(s) can be found in the article/Supplementary Material.
