# Supplementary material for: Unveiling the kinetic versatility of aryl-alcohol oxidases with different electron acceptors
Source: Front Bioeng Biotechnol. 2024 Aug 5;12:1440598. doi: 10.3389/fbioe.2024.1440598 (PMC11330772; doi:10.3389/fbioe.2024.1440598)
Supplement: Supplementary file 1 [file DataSheet1.PDF]

Supplementary information

Unveiling the kinetic versatility of aryl alcohol oxidases with different electron acceptors

Ana Serrano[1†#], Paula Cinca-Fernando[2,3#], Juan Carro[1], Adrián Velázquez-Campoy[2,3,4,5], Marta Martínez-Júlvez[2,3], Ángel T. Martínez[1*] and Patricia Ferreira[2,3*]

[1] Centro de Investigaciones Biológicas Margarita Salas, CSIC, Madrid, Spain
[2] Departamento de Bioquímica y Biología Molecular y Celular, Facultad de Ciencias, Universidad de Zaragoza, Zaragoza, Spain
[3] Instituto de Biocomputación y Física de Sistemas Complejos, BIFI (GBsC-CSIC Joint Unit), Universidad de Zaragoza, Zaragoza, Spain
[4]Institute for Health Research Aragon (IIS Aragon), Zaragoza, Spain
[5]Biomedical Research Networking Center in Hepatic and Digestive Diseases (CIBERehd), Madrid, Spain
[†]Current address: Certest Biotec SL, San Mateo de Gállego, Zaragoza, Spain
[#]These authors contributed equality

[*]Correspondence:
Ángel T. Martínez
atmartinez@cib.csic.es
Patricia Ferreira
ferreira@unizar.es

Supplementary information includes: **Figures S1-S6**; **Table S1**

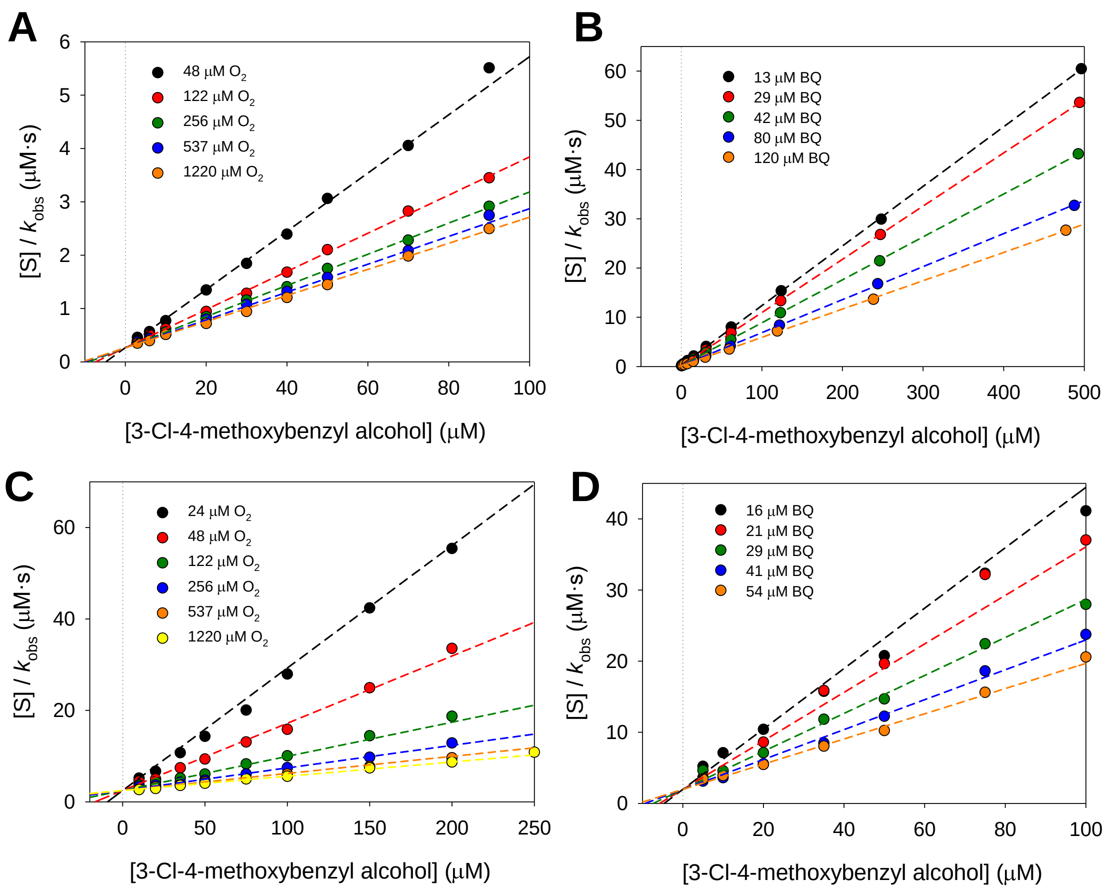

Figure S1. Hanes-Woolf plots for bi-substrate kinetics with 3-Cl-4-methoxybenzyl alcohol. Kinetics for *Pe*AAO (**A**, **B**) and *Ba*AAO (**C**, **D**) with O_2 in **A** and **C** and BQ in **B** and **D**. Data were measured by varying the concentrations of both alcohol substrate and electron acceptor in 50 mM sodium phosphate pH 6.0 at 25 °C. The vertical dotted line represents x = 0.

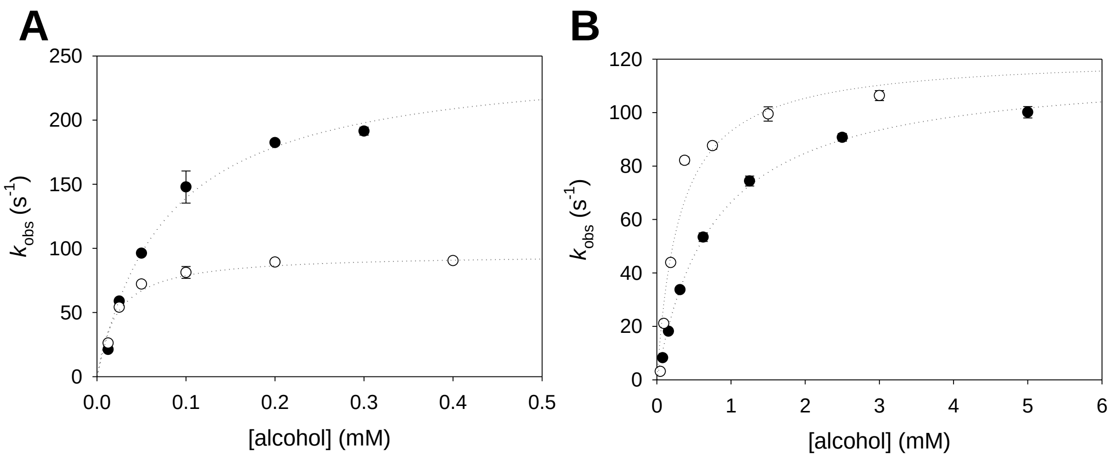

Figure S2. Reductive half-reaction. Plots of the observed rate constants (k_{obs}) of flavin reduction for *Pe*AAO (**A**) and *Ba*AAO (**B**) (~10 µM) with varying concentrations of 4-Methoxybenzyl alcohol (closed circles) and 3-Cl-4-methoxybenzyl alcohol (open circles). Data were measured in an anaerobic stopped-flow spectrophotometer in 50 mM sodium phosphate pH 6.0 at 25 °C. Lines represent the fits of experimental data to equation 3. Means and standard deviation were calculated from triplicates.

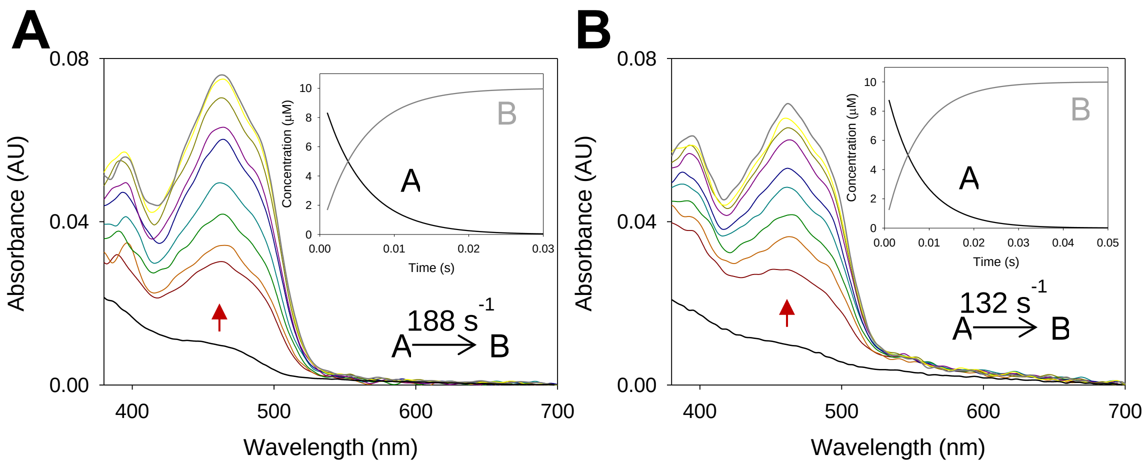

Figure S3. Time course of reoxidation of *Ba*AAO with O_2. (**A**) Spectra of reduced *Ba*AAO (~10 μM) by 4-Methoxybenzyl alcohol and measured at 0.001, 0.003, 0.004, 0.005, 0.008, 0.01, 0.015, 0.025 and 0.03 s after mixing with O_2 (1.6 mM). (**B**) Spectra of reduced *Ba*AAO (~10 μM) by 3-Cl-4-methoxybenzyl alcohol and measured at 0.001, 0.004, 0.005, 0.007, 0.01, 0.015, 0.02, 0.025, and 0.05 s after mixing with O_2 (1.6 mM). The red arrows indicate the direction of spectral change observed when the time is increased. Black lines correspond to the reduced enzyme by the alcohol before mixing with O2 and gray lines correspond to the last spectrum after total reoxidation. Reactions were performed in 50 mM sodium phosphate, pH 6.0 at 12 ºC. Insets show evolution of species A (black line) and B (gray line) after data fitting to a one-step process.

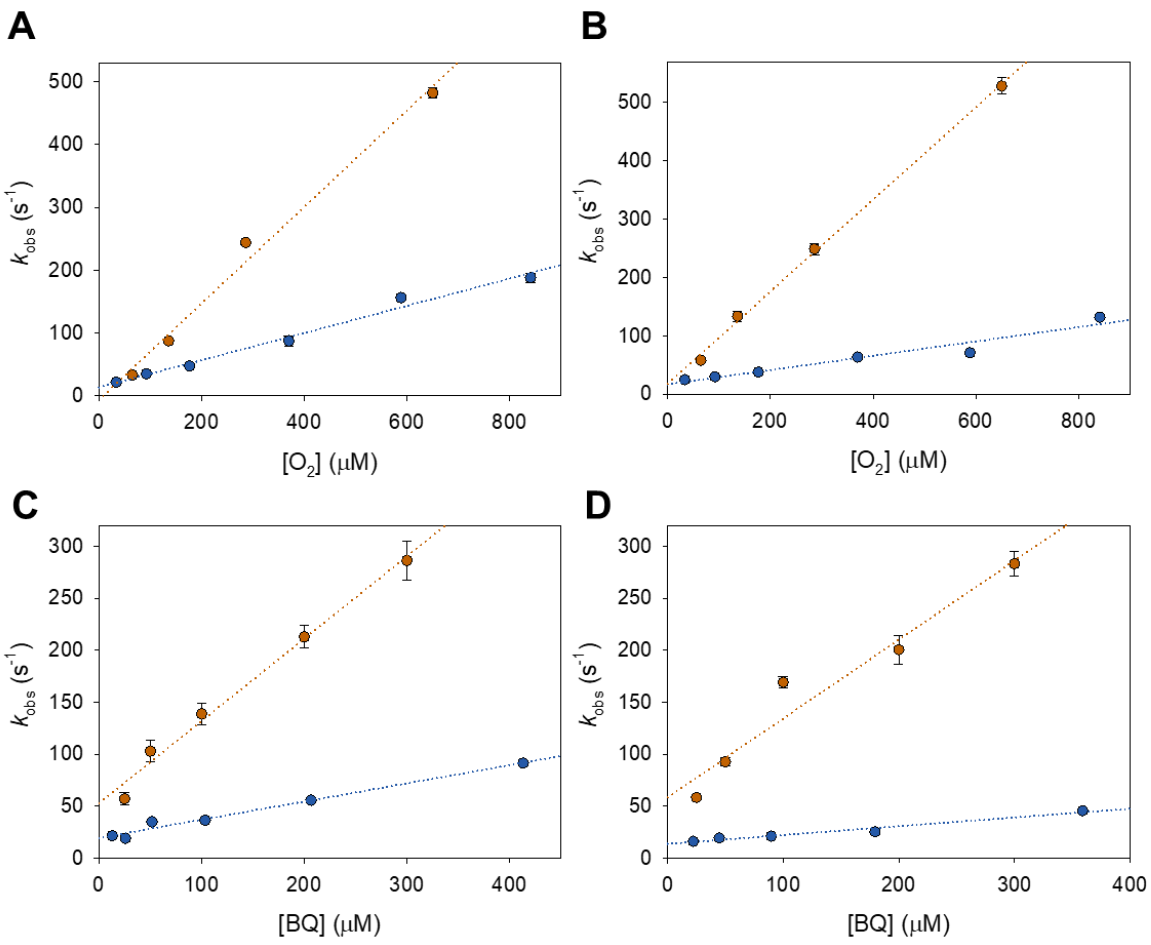

Figure S4. Oxidative half-reaction. Plots of the observed rate constants (k_{obs}) of flavin oxidation for *Pe*AAO (orange) and *Ba*AAO (blue), reduced with either 4-Methoxybenzyl alcohol (**A** and **C**) or 3-Cl-4-methoxybenzyl alcohol (**B** and **D**), and as a function of O_2 concentration (**A** and **B**) or BQ concentration (**C** and **D**). Data were measured in an anaerobic stopped-flow spectrophotometer in 50 mM sodium phosphate pH 6.0 at 12 °C. Lines represent the fits of experimental data to equation 4. Means and standard deviation were calculated from triplicates.

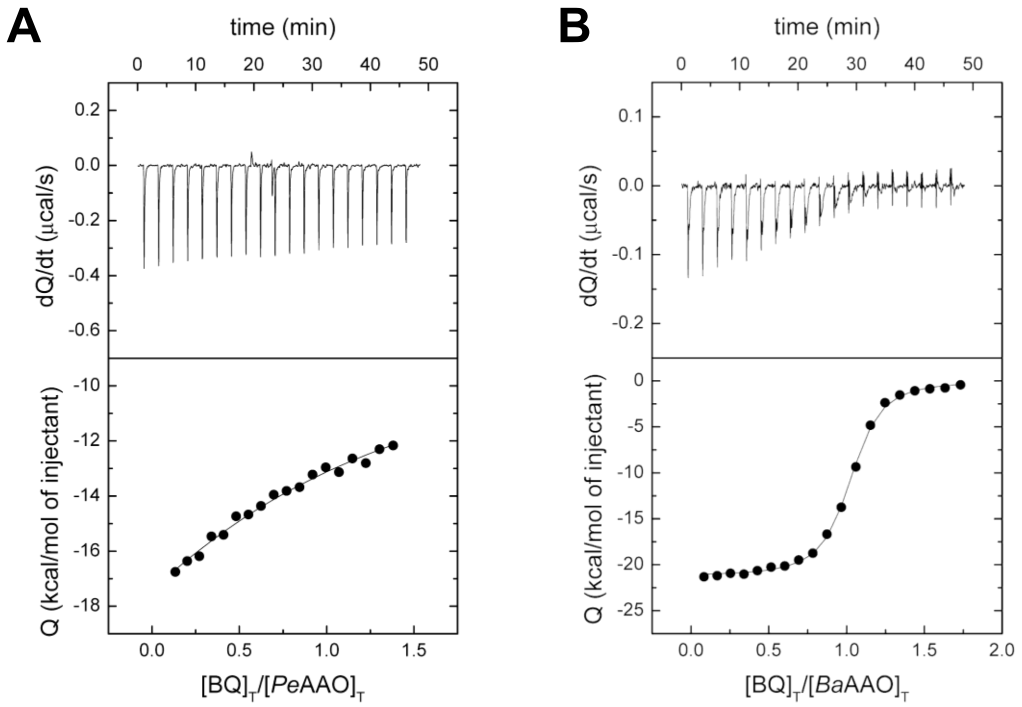

Figure S5. Calorimetric titrations for binary interactions of (**A**) *Pe*AAO:BQ, and (**B**) *Ba*AAO:BQ. Measurements were performed in 50 mM Tris-HCl pH 7.0 at 25 °C. The upper panels show the thermograms for the interactions, whereas the lower panels show the corresponding binding isotherms with ligand-normalized integrated heats. Data were fitted to a home-derived model for a single ligand binding site (continuous lines in binding isotherms).

A

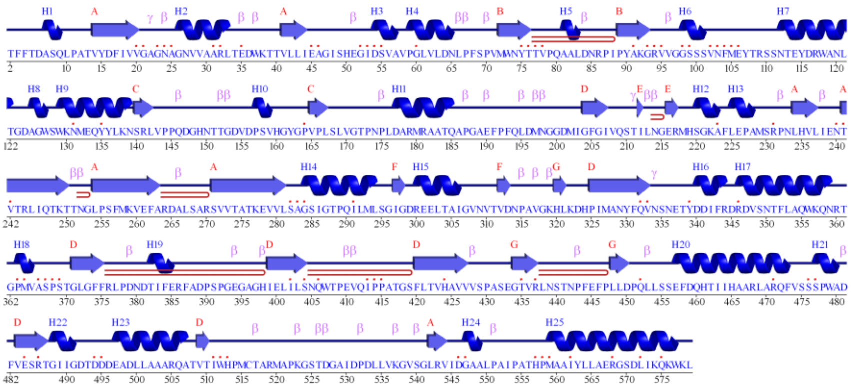

B

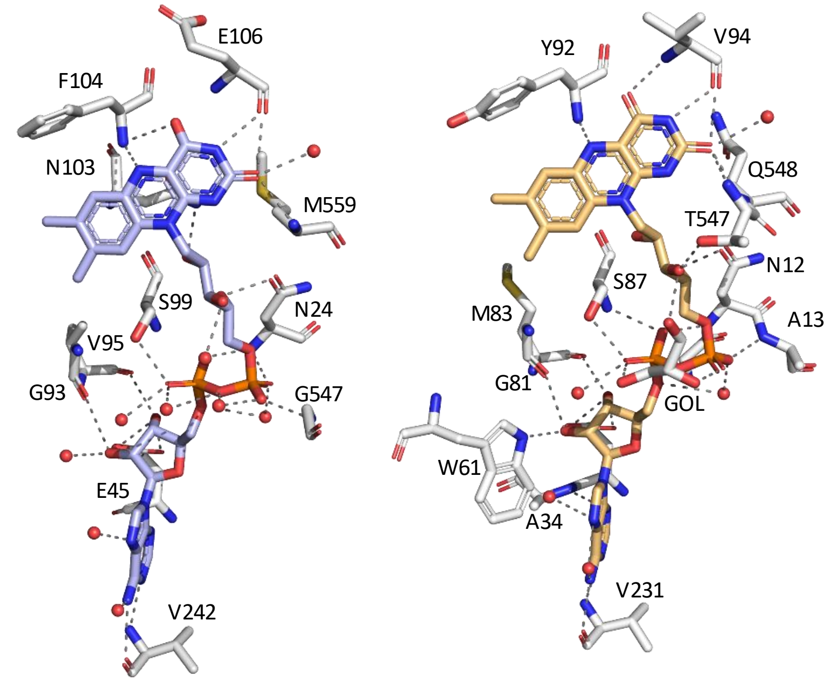

Figure S6. (A) Schematic "wiring diagram" of the *Ba*AAO:4-Methoxybenzoic acid complex´s secondary structure. Scheme includes strands (light blue arrows), helices (deep blue springs), and other motifs in red: β-turns (β), γ-turns (γ) and β-hairpins (⊂⊃). Residues in contact with ligands are highlighted with red points above. The figure was generated by PDBsum (1) for PDB entry 9AVH. **(B)** Structural details of FAD-AAO interactions in *Ba*AAO (PDBid 9AVH, left, blue carbons) and *Pe*AAO (PDBid 5OC1, right, orange carbons). H-bonds are indicated by dashed lines, and residues are labeled.

Table S1. Selected similar 3D structures to that of the *Ba*AAO:4-Methoxybenzoic acid complex*.

	% Identity	Amino acid overlap	PDB code	Protein name
1	48.0	563	5OC1	Aryl-alcohol oxidase from *Pleurotus eryngii* in complex with *p*-anisic acid
2	37.7	591	4H7U	Pyranose dehydrogenase from *Agaricus meleagris*
3	31.0	600	6ZE2	FAD-dependent oxidoreductase from *Chaetomium thermophilum*
4	30.2	592	7VKD	FAD-dependent glucose dehydrogenase from *Aspergillus oryzae*
5	30.1	592	4YNT	Glucose dehydrogenase from *Aspergillus flavus*
6	28.9	599	1CF3	Glucose oxidase from *Apergillus niger*
7	28.8	594	8BXL	Patulin synthase from *Penicillium expansum*
8	29.4	609	6XUT	Oligosaccharide dehydrogenase from *Pycnoporus cinnabarinus*
9	27.4	588	7AV4	C432C mutant of fatty acid photodecarboxylase from *Chlorella variabilis*
10	27.3	596	1GPE	Glucose oxidase from *Penicillium amagasakiense*
11	24.8	644	6H3G	Alcohol oxidase from *Phanerochaete chrysosporium*

*FASTA results from a search of *Ba*AAO sequence against all protein sequences in the PDB.

1. R. A. Laskowski, J. Jabłońska, L. Pravda, R. S. Vařeková and J. M. Thornton: PDBsum: Structural summaries of PDB entries. *Protein Sci*, 27(1), 129-134 (2018) doi:10.1002/pro.3289
